# Weed evolution: Genetic differentiation among wild, weedy, and crop radish

**DOI:** 10.1111/eva.12699

**Published:** 2018-09-29

**Authors:** Amanda Charbonneau, David Tack, Allison Lale, Josh Goldston, Mackenzie Caple, Emma Conner, Oz Barazani, Jotham Ziffer‐Berger, Ian Dworkin, Jeffrey K. Conner

**Affiliations:** ^1^ Genetics Michigan State University East Lansing Michigan; ^2^ Department of Biology Pennsylvania State University University Park Pennsylvania; ^3^ Kellogg Biological Station and Department of Plant Biology Michigan State University Hickory Corners Michigan; ^4^ Rosenstiel School of Marine and Atmospheric Science Miami Florida; ^5^ Israel Plant Gene Bank Rishon LeZion Israel; ^6^ The Herbarium of the Hebrew University of Jerusalem Jerusalem Israel; ^7^ Department of Biology McMaster University Hamilton Ontario Canada

**Keywords:** adaptation, agriculture, ecological genetics, natural selection and contemporary evolution, population genetics—empirical

## Abstract

Approximately 200 weed species are responsible for more than 90% of crop losses and these comprise less than one percent of all named plant species, suggesting that there are only a few evolutionary routes that lead to weediness. Agricultural weeds can evolve along three main paths: they can be escaped crops, wild species, or crop‐wild hybrids. We tested these three hypotheses in weedy radish, a weed of small grains and an emerging model for investigating the evolution of agricultural weeds, using 21 CAPS and SSR markers scored on 338 individuals from 34 populations representing all major species and sub‐species in the radish genus *Raphanus*. To test for adaptation of the weeds to the agricultural environment, we estimated genetic differentiation in flowering time in a series of common garden experiments with over 2,400 individuals from 43 populations (all but one of the genotyped populations plus 10 additional populations). Our findings suggest that the agricultural weed radish *R. r. raphanistrum* is most genetically similar to native populations of *R. r. raphanistrum* and is likely not a feral crop or crop hybrid. We also show that weedy radish flowers more rapidly than any other *Raphanus* population or cultivar, which is consistent with rapid adaptation to the frequent and severe disturbance that characterizes agricultural fields.

## 1 INTRODUCTION

Modern farming provides a nearly ideal environment for fostering the rapid evolution of weeds. Plants growing in novel environments often evolve rapidly (Buswell, Moles, & Hartley, 2011) and human alterations of the environment often impose strong selection (Palumbi, 2001; Reznick & Ghalambor, 2001). In invasive species, rapid adaptation is also correlated with high levels of standing genetic variation, repeated introductions, and high levels of environmental disturbance (Sakai, Allendorf, Holt, & Lodge, 2001). Agricultural fields combine all of these factors. They are a created ecosystem unlike anything found in nature, where humans impose frequent and regular disturbance from tilling and harvesting, and contaminated seed stocks can repeatedly introduce large numbers of weed seeds. Understanding both the origins and adaptations of weeds could guide improvements in weed management (McNeill, 1976; Müller‐Schärer, Schaffner, & Steinger, 2004; Neve, Vila‐Aiub, & Roux, 2009; Stewart et al., 2009; Vigueira, Olsen, & Caicedo, 2012) as well as provide insight into approaches for preventing the evolution of future weeds (Higgins, Heikes, & Kempen, 1978).

There are three potential origin routes for agricultural weeds: crops going feral, wild populations invading fields, or crop‐wild hybridization (de Wet, 1966; de Wet & Harlan, 1975; Vigueira et al., 2012); and these differing histories may have detectably different phenotypic and genetic effects. Escaped crops, for example, may already be resistant to herbicides or able to survive in disturbed habitats (Vigueira et al., 2012). These different origins would also leave a distinct genetic signature at neutral markers. Weeds could have a wild origin, with either a wild population that was preadapted, or one that rapidly evolved to weediness. Here we would expect strong genetic resemblance between the weed and one or more native populations (Vigueira et al., 2012). Alternatively, the weeds could be crop‐wild hybrids, in which case we would expect the weeds to be a genetic mixture of cultivars and native plants with relatively high genetic diversity compared to the parental crop. Finally, if the weeds are most genetically similar to the cultivars at neutral markers, this would suggest that they descend from escaped crops. As feral crops would suffer from the founder effect of escaping cultivation after having undergone artificial selection, we might also expect weeds derived from crops to be less genetically variable than either wild invaders or hybrids (Vigueira et al., 2012).

Although these potential weed origins are widely discussed in the literature (Baker, 1974; Baker, 1991; de Wet, 1966; Dekker, 2011; Ellstrand et al., 2010, 2013; Gressel, 2005; Harlan, 1992; Vigueira et al., 2012), strong tests for most species are lacking, often due to a lack of information about wild relatives (Vigueira et al., 2012). Studies of weed origins can be challenging, as the genetic similarity of populations in weed‐crop‐wild triads, especially when combined with subsequent hybridization, can make it difficult to identify potential source populations for the weed (Ellstrand et al., 2010; Vigueira et al., 2012). Ellstrand et al. (2010) searched the literature specifically for cases where there was good evidence for highly domesticated ancestors being the ancestor of an agricultural weed and could find only 12 examples. We are unaware of similar systematic literature searches for weeds thought to have originated from wild species, although Vigueira et al. (2012) review a handful of possible examples. One of the better‐studied wild to weedy transitions is sunflower, *Helianthus annuus*, where across the USA weed populations were shown to be least differentiated from local wild populations compared to other weed populations (Kane & Rieseberg, 2008).

Phenotypic adaptations of agricultural weeds have also been relatively neglected, with the exception of adaptations to human control, particularly the evolution of crop mimicry and herbicide resistance (reviewed in Barrett, 1983; Neve et al., 2009; Vigueira et al., 2012). However, weeds must already be well‐adapted to agricultural habitats before they are problematic enough for humans to implement control practices. Some attention has been paid to the key adaptations of seed dormancy and shattering in the evolution of agricultural weeds from crop ancestors (Ellstrand et al., 2010; Vigueira et al., 2012), but there has been little work on the evolution of a shortened life‐cycle. In an agricultural setting, frequent and regular disturbances from plowing and harvesting likely exert a strong selection on weeds for rapid flowering and seed set (Barrett, 1983; Warwick & Stewart, 2005).

Weedy radish, *Raphanus raphanistrum* ssp. *raphanistrum*, is an emerging model for studying both rapid adaptation and weed evolution (Campbell, Snow, & Ridley, 2006; Campbell, Snow, Sweeney, & Ketner, 2009; Conner et al., 2011; Conner, Mills, Koelling, & Karoly 2014; Klinger, Elam, & Ellstrand, 1991; Ridley & Ellstrand, 2008; Sahli, Conner, Shaw, Howe, & Lale, 2008; Snow & Campbell, 2005) and is a member of one of the four major weed and crop families (*Brassicaceae*). Determining the origins of weedy radish is tractable because the genus *Raphanus* includes only three named species, all of which are self‐incompatible. The genus likely originated in the Mediterranean, as native populations exist only there (I. Al‐Shehbaz, pers. comm.). *R. raphanistrum* is divided into two subspecies, with *R. r. raphanistrum* including native Mediterranean populations as well as the globally distributed weed, and *R. r. landra* existing only as native Mediterranean populations. The crop *R. sativus* is divided into four major types—two root crops (European radish and Asian daikon) and two fruit crops (Oilseed and edible‐pod Rattail). *R. pugioniformis* is a little‐studied endemic of the eastern Mediterranean (Ziffer‐Berger, Hanin, Fogel, Mummenhoff, & Barazani, 2014). The relationships among these species are not well‐resolved (Ziffer‐Berger et al., 2014), but an analysis of cDNA sequence from one population of each of eight *Raphanus* taxa (not including *R. pugioniformis*) provided strong support for the monophyly of the crop cultivars, as well as monophyly of native and weedy *R. r. raphanistrum* (Shen et al., 2013).

While native *Raphanus* is found only near the Mediterranean, weedy populations are found on every continent except Antarctica (Holm, 1997). Weedy *R. r. raphanistrum* is a common contaminant of small grain fields and is considered one of the world's worst agricultural weeds (e.g., Blackshaw, Lemerle, Mailer, & Young, 2002; Culpepper, 2012; Holm, 1997; Schroeder, 1989; Warwick & Francis, 2005; Webster & Macdonald, 2001). Weedy radish has been used extensively in ecological and evolutionary studies, particularly for plant‐insect interactions (Agrawal, Conner, Johnson, & Wallsgrove, 2002; Bett & Lydiate, 2003; Conner, 2002; Conner, Franks, & Stewart, 2003; Devlin & Ellstrand, 1990; Irwin, Strauss, Storz, Emerson, & Guibert, 2003; Lehtilä & Strauss, 1999; Malik, 2009; Mazer & Schick, 1991; Morgan & Conner, 2001; Snow, Uthus, & Culley, 2001; Stanton, Snow, & Handel, 1986) and has genomic resources (Moghe et al., 2014) that make it an ideal study system to answer questions about both the origins and adaptations of agricultural weeds. Studies have shown that weedy radish lacks a signal of isolation by distance (Barnaud, Kalwij, Berthouly‐Salazar, McGeoch, & Jansen van Vuuren, 2013; Barnaud, Kalwij, McGeoch, & van Vuuren, 2013; Kercher & Conner, 1996), likely due to human‐mediated movement of large numbers of seeds long distances as a contaminant of grain and other agricultural products (Cheam, 2006; Holm, 1997; Snow & Campbell, 2005).

Previous work has shown that eight populations of weedy radish flowered much more quickly than one native *R. r. raphanistrum* population in a greenhouse common garden (Sahli et al., 2008). Similarly, five weedy radish populations flowered faster than five root‐crop radish cultivars in greenhouse (Hegde, Nason, Clegg, & Ellstrand, 2006) and field (Ridley & Ellstrand, 2008) common gardens. However, since flowering time has only been reported for one native population, and the phylogeny in Shen et al. (2013) was based on only a single population of each *Raphanus* taxon and did not include *R. pugioniformis*, neither the origin of weedy radish nor whether the weeds have evolved earlier flowering is clear. We estimated genetic differentiation for flowering time and molecular markers for all named species and subspecies in the genus *Raphanus*, including a total of 15 populations from all three wild taxa from the native range, eight weedy *R. r. raphanistrum* populations from outside the native range, and 21 crop cultivars from all four groups (Supporting Information Table S1), to address two questions. First, did weedy radish originate from native *R. r. raphanistrum* as previous work suggests, or instead as an escaped crop or a crop‐wild hybrid? Second, is there evidence that the weeds have evolved more rapid flowering relative to the rest of the genus, again as hypothesized based on previous work?

## 2 MATERIALS AND METHODS

### 2.1 Populations

Eleven *R. r. raphanistrum* populations from the native Mediterranean range were included, with six from the western part of the range (collected in Spain or France) and five eastern (collected in Israel). Only one of these populations (AFFR from southern France) was collected in an agricultural field; all but one of the rest were from human‐disturbed nonagricultural habitats (Supporting Information Table S1). We used eight populations from outside the native range; all but one were collected as weeds of agricultural fields in the USA, Europe, and Australia, and the one exception (MAFI from Finland) was collected in an agricultural landscape. Thus, *R. r. raphanistrum* exists primarily as an agricultural weed outside its native Mediterranean range, and our conclusions about the evolution of the weeds are based on comparing native to non‐native populations. Three populations of *R. r. landra* from Spain and one *R. pugioniformis* population from Israel were also included, as were twenty‐one crop varieties purchased from seed companies (Supporting Information Table S1). The natural populations were collected by a variety of individuals using a variety of methods, including collecting from all fruiting individuals in small populations and collecting from transects or grids in large populations; in all cases, the goal was to sample the genetic variation of each population in an unbiased manner.

### 2.2 Genotyping

To assay patterns of neutral genetic differentiation among these populations, we used a panel of 13 CAPS (cleaved amplified polymorphic sequences) in addition to the 8 SSR (microsatellite) markers from Sahli et al. (2008). To create the CAPS markers, cDNA sequencing of seven lines of *Raphanus* populations and cultivars (Moghe et al., 2014) was used to assemble and align line‐specific contigs against each other (Supporting Information Table S2). None of the markers are closely linked, with the closest pair being 6 cM apart (Supporting Information Table S2). Genotyping was completed on 10 randomly sampled plants from each of 34 populations for a total of 338 individuals (Supporting Information Table S1; two cultivars, ESNK and RACA had 9). Some of the non‐native and crop cultivars were left out of the genotyping to improve balance across the different groups and save genotyping costs. For non‐native *R. r. raphanistrum,* we left out three populations in regions (North America, Scandinavia, and Australia) that were represented by other populations. For the crops, we left out less‐common cultivars in three of the crop groups (Supporting Information Table S1). Two microsatellites, Na10H06 and Na14E08, had fairly high numbers of missing genotypes (26% and 31%, respectively); however, these were concentrated among crop cultivars, and dropping these markers did not have a qualitative impact on our results, so the data presented here include all markers. Standard summary statistics were computed using the R (v 3.2.2, R Core Team, 2015) packages “adegenet” (v.2.0.1, Jombart, 2008; Jombart & Ahmed, 2011) and “pegas” (v.0.10, Paradis, 2010).

### 2.3 Quantitative analyses of marker variation

First, we used the R package hierfstat (v 0.04‐22, Goudet & Jombart, 2015) to calculate pairwise *F*_ST_ among all populations and then clustered populations using the Euclidean distances among these pairwise *F*_ST_ values to test the three hypotheses for weed origins. We then created three analysis of molecular variance (AMOVA) models to further test each of the three hypotheses. AMOVA is analogous to a nested ANOVA, performing a hierarchical analysis of marker variance, estimating the percent of molecular variance accounted for by each level of the nested sampling hierarchy as well as *ϕ*_ST_, an *F*_ST_ analogue summarizing differentiation among groups or the individual populations nested within each group (Excoffier, Smouse, & Quattro, 1992).

To test whether weeds are likely to be feral crops, we used AMOVA to analyze a subset of the data that included only *R. sativus* and non‐native *R. r. raphanistrum*. In this model, population of origin was nested in group, where group was either crop (*R. sativus*) or weed (non‐native *R. r. raphanistrum*). In a parallel fashion, to test whether weeds are likely derived from native *R. r. raphanistrum*, we analyzed a subset of the data that included only native and non‐native *R. r. raphanistrum*, which formed the two groups. Finally, to test whether weeds are more closely related to native *R. r. landra*, we analyzed a subset of the data that included only *R. r. landra* and non‐native *R. r. raphanistrum,* which again formed the two groups in the analysis. We obtained *p* values for all tests using 500 permutations.

All AMOVA were run using the ade4 reimplementation from the “poppr” package (v2.6.1, Kamvar, Tabima, & Grünwald, 2014) for R (v 3.2.2, R Core Team, 2015). In all cases, we used raw pairwise distances, set “filter” to TRUE, did not calculate individual variance by haplotype, and ignored missing data, as removing missing values had no qualitative effect on the results. Graphs were produced using custom scripts and ColorBrewer (v.1.1‐2, Neuwirth, 2014).

### 2.4 Visualization of marker variation

To complement the AMOVA and *F*_ST_ analyses, we also performed a principal components analysis to visualize the patterns of marker variation in the genus. SmartPCA (v.13050—from the program Eigensoft 6.0.1) (Patterson, Price, & Reich, 2006) was used to perform an eigen decomposition optimized for genomic data to rotate the data onto a set of orthogonal axes defined by the amount of variation explained. Although this method rotates, rather than clusters the data, it can reveal hidden data structure. SmartPCA was originally developed for datasets where the number of markers vastly exceeds the number of individuals genotyped, where performing a standard PCA would be difficult. Our dataset does not fit this expectation, as we have relatively few markers compared to individuals; however, SmartPCA still outperformed standard PCA. The SmartPCA algorithm tolerates missing genotypes, so we could use all individuals and markers in our analysis rather than dropping any with missing data. A standard PCA gave a qualitatively similar result, just with fewer usable data points (Supporting Information Figure S3).

As the SmartPCA algorithm is designed for biallelic markers, each SSR marker was expanded into several biallelic markers as described in Patterson et al. (2006), prior to analysis using a custom script. Experimentally determined linkage groups (http://radish.plantbiology.msu.edu/) were used as a proxy for chromosomes. Markers that could not be assigned to linkage groups were given unique chromosome numbers. The total number of linkage groups (7) plus singletons (3) was used as the chromosome number (Supporting Information Table S2); note that this is one more than the nine *Raphanus* chromosomes. SmartPCA analyses were run on the HPCC at Michigan State University and results plotted using custom bash and R scripts (v 3.2.2, R Core Team, 2015).

### 2.5 Flowering time common gardens

To test for genetic differentiation in flowering time, we combined data from eight common garden experiments performed over a period of 11 years and including a total of 2,441 plants (Table 1). Five of these experiments (G‐03, G‐04, F‐05, G‐10, and G2‐13) had a relatively large number of individuals per population (mean = 49), but a small number of populations per experiment (range 4–9), and while there was overlap between experiments, each had a unique combination of populations. To complement results from these trials, we also included data from three additional common garden experiments (two field, one greenhouse) with both more populations each, and more overlap between populations (F‐12, F‐13, and G1‐13). Two experiments (G‐04, F‐05) used seeds from full‐sibling families as described in Sahli et al. (2008); we did not account for this in the analysis due to the complexity of the models used and the fact that the other experiments also include natural and unknown family structure.

**Table 1:** Summary of the eight flowering time common garden experiments, which took place at one of two sites in Michigan over a period of 11 years

Experiment	Year	Location	Field GH	NPops	NperPop	TotalN	References
G‐03	2003	KBS	GH	9	22–46	306	Parentals (Sahli et al., 2008)
G‐04	2004	KBS	GH	9	58–142	877	Offspring (Sahli et al., 2008)
F‐05	2005	KBS	Field	6	64–88	442	Offspring (Sahli et al., 2008)
G‐10	2010	KBS	GH	4	8–22	55	
F‐12	2012	KBS	Field	13	7–10	127	
F‐13	2013	MSU	Field	23	10	229	
G1‐13	2013	KBS	GH	15	10	150	
G2‐13	2013	KBS	GH	9	14–30	254	

*Note*

NPops is number of populations in each experiment. Number of individuals in each population is given as NperPop. TotalN is the total number of individuals in that experiment.

In all experiments, locations of individual plants were randomized with respect to population, and seeds were removed from pods prior to planting to minimize variability in germination times. All plants were monitored daily for germination and until they flowered or died without flowering. In the field experiments, plants were left in the field to over‐winter and monitored for flowering the following spring. In the greenhouse experiments, 225 *R. raphanistrum* plants from the three *R. r. landra* and one of the native populations (MAES) did not flower in 82–120 days, and these received a vernalization treatment. We recorded germination date and date of first flower on all plants; the difference between these dates is our measure of flowering time. Plants were watered as needed.

Phenotypic data from all eight experiments were concatenated for analysis as a single dataset. Only a small subset of plants from four native populations were subjected to vernalization (see above). Similarly, although most populations are represented in multiple experiments, the datasets are not balanced. However, if we examine flowering time in the three populations (one weedy, one native *R. r. raphanistrum* and one *R. r. landra*) that were included in four or five experiments including both greenhouse and field, we see that there is much more variance in flowering time among populations than among experiments within populations (Supporting Information Figure S7). Still, we accounted for these aspects of the dataset by analyzing the flowering time data using two mixed models in a Bayesian framework, similar to a modern meta‐analysis. The first model includes only *R. r. raphanistrum* plants, that is, those with little or no vernalization requirement. The second includes only native *Raphanus*, which have variable vernalization requirements. To determine whether vernalization time had an effect on parameter estimates, we also ran both models with and without vernalization (*ν*) as a fixed effect. In all plots of modeled values, both estimates are shown. This method resulted in two reasonably balanced datasets and provides two estimates for native *R. r. raphanistrum*. We also provide un‐modeled population level estimates of both flowering time and vernalization requirement.

We modeled days to flower as a function of geographic origin to verify the differences in flowering time between native and non‐native *R. r. raphanistrum* populations reported by Sahli et al. (2008), but with multiple native populations. We also tested for differences between native populations from eastern and western Mediterranean. For this analysis, we used a subset of the data that only included *R. r. raphanistrum* populations (Supporting Information Table S1) and controlled for a number of covariates. This model took the form of:

(1) $$\begin{array}{c} f_{i}∼N\left(\beta_{0}+\beta_{r}\chi_{1,i}+\beta_{m}\chi_{2,i}+\beta_{\mathrm{DOYGS}}\chi_{3,i}+\beta_{\nu }\chi_{4,i}+\eta_{j[i]}+\eta_{k[i]},\sigma_{f}^{2}\right)\\ \text{with}\\ \eta_{j}∼N\left(0,\sigma_{\mathrm{experiment}}^{2}\right)\\ \text{and}\\ \eta_{k}∼N\left(0,\sigma_{\mathrm{population}}^{2}\right) \end{array}$$

where *f* is the number of days from germination to flowering for plants that flowered, the number of whole days from germination to bolting for plants that bolted but did not flower, and the number of whole days from germination until the last day of monitoring in the fall, plus one, for plants that survived without flowering until this date but did not survive the winter. The purpose here is to not bias the analysis by excluding plants that never flowered; in the results we also present flowering time data for only those plants that flowered as well as the percent of plants that flowered. *r* is the geographic region of origin (western or eastern Mediterranean, or outside this native range). *m* is a factor representing whether the seed producing that individual was collected directly from the population of origin or generated in the greenhouse. DOYGS is the day of year that the plant germinated (1–365) scaled such that 1 is the spring equinox in the northern hemisphere. *ν* is the number of days each plant was vernalized, if any. The random effect *η*_*j*_ is the experiment that each plant was in, and accounts for year‐to‐year variation as well as field versus greenhouse and other variation in experimental protocols. The random effect *η*_*k*_ accounts for variance among populations within geographic regions.

To look for differences in flowering time among *Raphanus* species and sub‐species from within the native range, we used a subset of the data that included only populations collected from within the Mediterranean region. This model took the form of:

(2) $$\begin{array}{c} f_{i}∼N\left(\beta_{0}+\beta_{u}\chi_{1,i}+\beta_{m}\chi_{2,i}+\beta_{\mathrm{DOYGS}}\chi_{3,i}+\beta_{\nu }\chi_{4,i}+\eta_{j[i]}+\eta_{k[i]},\sigma_{f}^{2}\right)\\ \text{with}\\ \eta_{j}∼N\left(0,\sigma_{\mathrm{experiment}}^{2}\right)\\ \text{and}\\ \eta_{k}∼N\left(0,\sigma_{\mathrm{population}}^{2}\right) \end{array}$$

where *u* is a factor representing species or sub‐species designation (*R. r. raphanistrum*,* R. r. landra*, and *R. pugioniformis*), and the other terms are as in the previous model.

All phenotypic models were run using the “MCMCglmm” package (v.2.2, Hadfield, 2010) for R (v 3.2.2, R Core Team, 2015). Graphs were produced using custom scripts and ColorBrewer, (v.1.1‐2, Neuwirth, 2014). Scripts and all data required for all analyses are available via the authors Github page: https://github.com/ACharbonneau/creepy-barnacle and on Dryad https://doi.org/10.5061/dryad.tc651j5.

## 3 RESULTS

### 3.1 Marker data are consistent with the hypothesis that weeds evolved from a native *R.r. raphanistrum* ancestor

**Figure 1:**
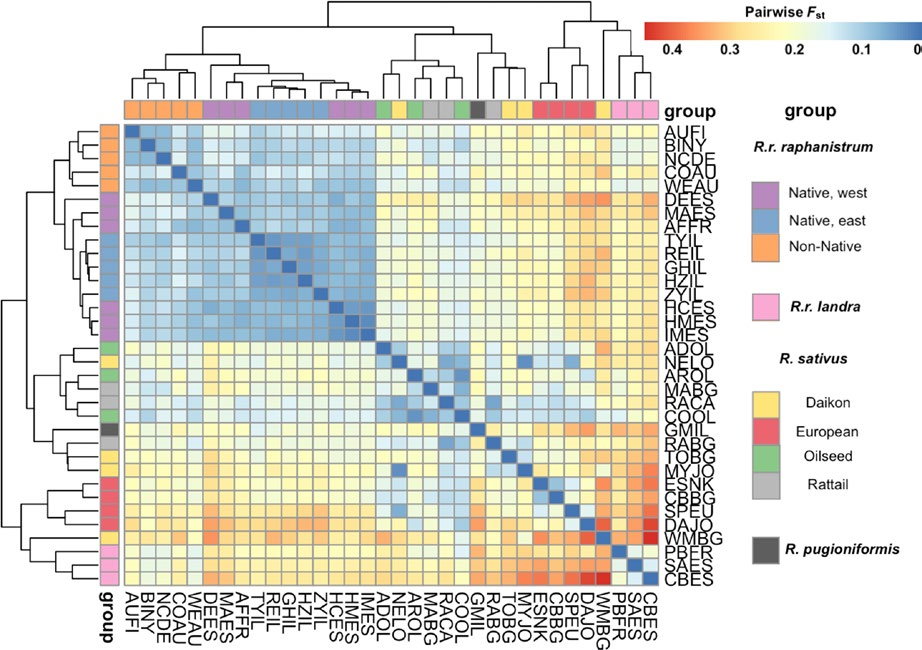
Pairwise *F*_ST_ calculated for all 21 markers, and clustered by Euclidean distance. Populations are colored along the axes to match the putative groups from the SmartPCA (Figure 2) analyses; for population codes on the other axes, see Supporting Information Table S1

The *F*_ST_ analyses show that the lowest levels of differentiation across the genus *Raphanus* are between the non‐native and native *R. r. raphanistrum* (Figure 1). Similarly, the AMOVA hierarchical *ϕ*_ST_ values were lower when the non‐natives were tested against native *R. r. raphanistrum* compared to the tests against crops or *R. r. landra* (Table 2). While the non‐natives are most similar to native *R. r. raphanistrum* based on the hierarchical *ϕ*_ST_, the AMOVA shows statistically significant differentiation between the non‐natives and each of the other three groups.

**Table 2:** Results of three AMOVAs

Hierarchical level of model	*ϕ*_ST_	% variance	*p* value
Weed versus crop			
Between groups	0.13	13.2	0.004
Between populations within groups	0.39	33.5	0.002
Within populations	0.47	53.4	0.002
Weed versus native *R. r. raphanistrum*			
Between groups	0.10	10.3	0.002
Between populations within groups	0.13	11.4	0.002
Within populations	0.22	78.3	0.002
Weed versus *R. r. landra*			
Between groups	0.21	20.6	0.024
Between populations within groups	0.25	19.9	0.002
Within populations	0.41	59.5	0.002

*Note*

Weed versus crop tests whether all non‐native *R. r. raphanistrum* as a group are significantly different from all *R. sativus* as a group. Similarly, weed versus native tests whether all non‐native *R. r. raphanistrum* as a group differ from all native *R. r. raphanistrum* as a group. Weed versus *R. r. landra* compares all non‐native *R. r. raphanistrum* as a group to all *R. r. landra* as a group. In all three models, populations were nested in groups, and % variance indicates the amount of variation accounted for by each hierarchical level of that model. Note that there is evidence for population structure at within groups in all three models.

**Figure 2:**
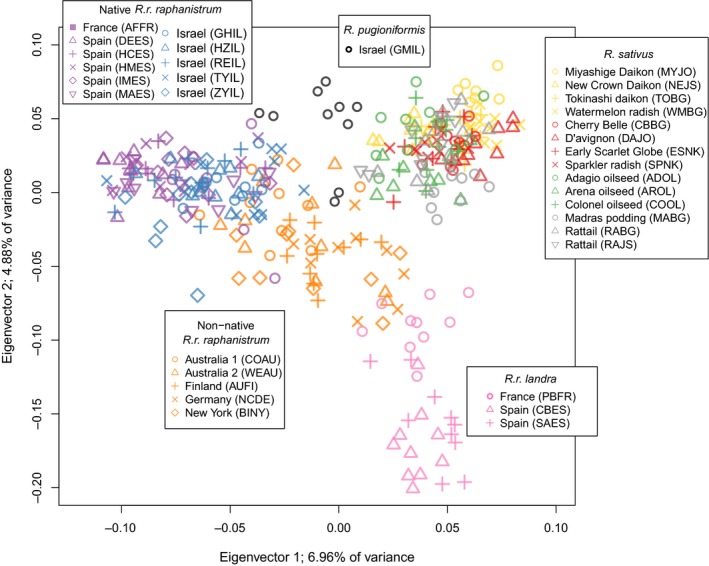
Smart PCA plot of the first two eigenvectors of a principal components analysis of 34 *Raphanus* populations genotyped at presumed neutral markers. Each point is an individual, and each population is represented by 10 individuals except two *R. sativus* cultivars (ESNK and RACA), which have nine. See Supporting Information Table S1 for population abbreviations

The PCA results (Figure 2) are consistent with the *F*_ST_ and AMOVA results and with taxonomic designations for the genus. All groups formed distinct clusters on the first two axes, with the non‐native populations in the center of the other groups and overlapping substantially only with native *R. r. raphanistrum*.

### 3.2 Non‐native range *R. r. raphanistrum* flower more rapidly

**Figure 3:**
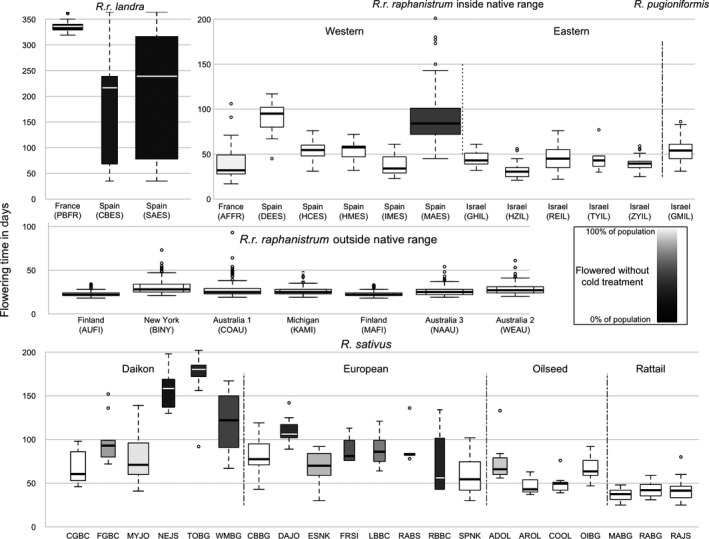
Raw Flowering times in *Raphanus*. Medians, quartiles, and outliers for raw days from germination to first flower for each population are shown, with shading to denote the proportion of plants that flowered without experiencing vernalization. Boxplot widths are a function of number of individuals per population, with wider plots indicating more individuals. Maximum: AUFI (*N* = 250); minimum: RBBC (*N* = 3), total = 2,054. Note that *Y*‐axis scales are the same except for *R. r. landra*, which has much longer flowering times. None of *R. r. raphanistrum* populations from outside the native range required vernalization

We found that non‐native radish consistently flowers more quickly than any other *Raphanus*. In model 1, non‐natives flowered about 58 days earlier than the Western range *R. r. raphanistrum*, and about 24 days earlier than the Eastern populations (Figure 3 and Supporting Information Figure S4). The native *R. r. raphanistrum* population with the fastest flowering time (AFFR) was the only one collected from an active agricultural field, while the slowest flowering (DEES) was the only one collected from an undisturbed habitat (Supporting Information Table S1). *R. r. landra* populations took even longer to flower. In model 2, they required an additional 76–139 days to flower over the average native *R. r. raphanistrum* plant, depending on whether vernalization time was included as a fixed effect (Supporting Information Figure S5). Not surprisingly, on average the root crops (daikon and European) flowered more slowly than the crops that are used for their fruits (oilseed and rattail), as flowering causes resources stored in the roots to be reallocated to the fruits.

There was considerable variation in flowering times among the *R. r. landra* and *R. sativus* populations (Figure 3, Supporting Information Figures S5 and S6). For example, although the *R. r. landra* populations cluster by genetic markers, the French population was slower to flower (due to an absolute vernalization requirement) compared to populations collected in Spain (Figure 3 and Supporting Information Figure S5); and the latter had much higher within‐population variation in flowering time than any of the other populations studied. In contrast, flowering time estimates for the weeds are consistently rapid with little within‐population variation (Figure 3 and Supporting Information Figure S6). Although this study spanned several years and experiments, most of the within‐population variation we observed is consistent across studies. Any given population has very similar flowering time distributions regardless of the time or location of the experiment, and the native *R. r. landra* population (SAES) consistently had a bi‐modal distribution (Supporting Information Figure S7) due to most individual SAES plants requiring vernalization.

## 4 DISCUSSION

We assayed a diverse set of populations across the genus *Raphanus*, to address two interconnected questions concerning the evolution of weedy radish. First, what is the likely origin of the weeds; and second, has weedy radish evolved rapid flowering in comparison with its progenitors?

### 4.1 Origins of radish as an agricultural weed

There are three main pathways to agricultural weediness; feral crops, wild invaders, and hybridization, either wild‐wild or wild‐crop. All our results are most consistent with the hypothesis that the weeds evolved from a native *R. r. raphanistrum* ancestor, in agreement with taxonomic designation of the weeds as members of this subspecies. *F*_ST_ analyses clearly cluster weedy and native members of this subspecies together, and while the AMOVA found that weedy and native *R. r. raphanistrum* are significantly differentiated, they are less differentiated than the weeds are from either the crop *R. sativus* or the other native subspecies *R. r. landra* (Table 2). This conclusion is in agreement with a previous phylogenetic analysis based on eight transcriptomes, which found that weedy *R. r. raphanistrum* and native *R. r. raphanistrum* are sister taxa, and that both are more closely related to the other native *Raphanus* than to any cultivar (Shen et al., 2013).

While our results are all consistent with native *R. r. raphanistrum* as the ancestor of the weeds, we cannot rule out introgression from other *Raphanus* taxa. Introgression is suggested both by the central position of the non‐native populations relative to all the other *Raphanus* groups in the PCA (Figure 2), as well as by the significant genome‐wide differentiation between the non‐native and native *R. r. raphanistrum* in the AMOVA (Table 2). The differentiation between non‐native and native could also have been caused by drift early in weed evolution before they spread across the globe; however, the weeds have the highest expected heterozygosities of all the groups tested (Supporting Information Table S8), consistent with introgression and inconsistent with strong effects of drift. Introgression with native *R. r. landra* could have occurred in the Mediterranean early in the evolution of the weeds, and there are ample current opportunities for weeds and crop radish to hybridize. The California invasive wild radish is the product of weedy and crop hybridization (Hegde et al., 2006; Panetsos & Baker, 1967), and weed‐crop hybridization has occurred elsewhere (Snow & Campbell, 2005). As the marker density of our data is relatively low, we would be unable to resolve small scale introgression followed by strong selection for adaptive alleles. Resolving patterns of past introgression is difficult in general, and strong evidence would require a much larger set of genomic markers.

In cases where the origin of agricultural weeds are known, researchers have frequently found them to be escaped crops (Ellstrand et al., 2010; Vigueira et al., 2012); however, our results do not support this. The crops are significantly different from the weeds in the AMOVA with a higher *ϕ*‐ST value than the weed‐native *R. r. raphanistrum* comparison (Table 2), and the latter two groups form a distinct cluster separate from the crops. These results are inconsistent with the crop origin theory but are consistent with previous work that found no shared chloroplast haplotypes between crop and weed populations (Ridley, Kim, & Ellstrand, 2008). Additionally, weeds resulting from de‐domestication are expected to have very low genetic diversity (Vigueira et al., 2012), but non‐natives in our study have the highest expected heterozygosity of any group (Supporting Information Table S8). This does not seem to be due to pooling genetically differentiated populations, as three of the five weed populations we genotyped also have the highest expected heterozygosites that we measured, and the weedy populations clustered together in our genetic analyses. Empirical work also suggests that newly escaped radish cultivars would make poor weeds; Campbell and Snow (2009) found no evidence that *R. sativus* could establish feral populations without introgression from weedy radish and were unable to artificially select for greatly reduced flowering time in the Red Silk cultivar. While there are some reports of feral crop radish in the literature, it seems likely that these are actually hybrids (Snow & Campbell, 2005). Taken together, these results are inconsistent with an “escaped crop” or “crop hybrid” origin for weedy radish, but introgression of crop genes into weedy radish is a possibility.

### 4.2 Evolution of faster flowering in weedy radish

Although non‐native *R. r. raphanistrum* populations are genetically similar to native *R. r. raphanistrum*, they flower much faster (25–58 days earlier flowering on average, Model 1). Weedy radish flowers much more rapidly and uniformly than any of the other *Raphanus* taxa, and the weeds never require vernalization (Figure 3). This lack of variation and decreased mean suggests that the weeds have undergone strong directional selection for flowering time. This supports the hypothesis that the weedy radish most likely arose from native *R. r. raphanistrum*, and subsequently evolved a faster flowering time. This more rapid flowering is in agreement with previous work, which has shown that non‐native *R. r. raphanistrum* flower faster than crop (Hegde et al., 2006; Ridley & Ellstrand, 2008) or native radish (Sahli et al., 2008).

This difference is especially striking in the raw data. We assayed days to flowering for non‐native radish from three continents in common garden experiments across a span of eleven years and eight experiments across three locations; however, we find almost no variation in the raw flowering time among or within the weed populations (Figure 3, Supporting Information Figure S7). This finding is somewhat surprising as phenotypic plasticity is expected to be a common feature of weeds (Baker, 1965) and invasives (Davidson, Jennions, & Nicotra, 2011; Richards, Bossdorf, Muth, Gurevitch, & Pigliucci, 2006) and flowering time has been found to be plastic in invasive plants, for example (e.g., Claridge & Franklin, 2002; Colautti & Barrett, 2010). As our common garden experiments were performed either in the summer or using summer conditions, it is possible that they were simply not different enough to trigger a plastic response.

In stark contrast to the phenotypic uniformity of the weeds, native range populations of both *R. r. landra* and *R. r. raphanistrum* varied in both days to flowering, and the need for vernalization. Interestingly, this also appears not to be a plastic response to our variable conditions. In native populations that were assayed in at least four common garden experiments, we find far more variation within experiments than between them, and that some native populations have reproducibly bimodal distributions of flowering times (Supporting Information Figure S7). Taken together, these data suggest that the native ancestors were likely more variable and slower‐flowering, at least partly due to a vernalization requirement, and this is consistent with the hypothesis of recent and rapid directional selection for shortened weed flowering time.

In summary, we found no evidence to support a crop origin for weedy radish, either by hybridization or exoferality. Non‐native *R. r. raphanistrum* likely descended from native *R. r. raphanistrum*, with possible introgression from other *Raphanus* taxa. All of these potential source populations have longer flowering times than we found among the weeds, which suggests that wild populations were not preadapted to field conditions and is evidence for rapid local adaptation to an agricultural habitat. Whether or not adaptation of weeds to agricultural conditions other than human control efforts is a more general phenomenon in requires additional studies comparing potential adaptive traits between agricultural weeds and their progenitors, as well as studies of present‐day selection on weeds in agricultural habitats.

## CONFLICT OF INTEREST

None declared.

## DATA ACCESSIBILITY

Seed stock information is in Supporting Information Table S1. Scripts are available at the authors Github page: https://github.com/ACharbonneau/creepy-barnacle. Data available from the Dryad Digital Repository: https://doi.org/10.5061/dryad.tc651j5.
