# Supplementary material for: Weed evolution: Genetic differentiation among wild, weedy, and crop radish
Source: Evol Appl. 2018 Sep 29;11(10):1964–74. doi: 10.1111/eva.12699 (PMC6231464; doi:10.1111/eva.12699)
Supplement: Supplementary file 1 [file EVA-11-1964-s001.pdf]

Table S1: All populations were included in at least one analysis of the paper: Genetic(G) or Phenotypic(P). Variety/Range refers to whether a wild population was collected inside (Native Range) or outside the Mediterranean region; or to the convariety name for cultivated varieties. Source refers to the individual who collected the source populations in the case of wild populations, or the company that cultivar seed was purchased from. Stock/Collection location is the seed company stock number for cultivars, and global position of the source population in the case of wild collected plants.

G	P	Population	Variety/Range	Habitat	Source Site	Stock/Collection location
R.r.landra						
X	X	PBFR	Native	Undisturbed	France	42°48'690N, 3°02'010E
X	X	CBES	Native	Undisturbed	Spain	43.4°N 4.23°W
X	X	SAES	Native	Undisturbed	Spain	43.49°N, 3.53°W
R.r.raphanistrum						
X	X	AFFR	Western	Ag field	France	43°08'260"N, 2°53'547"E
X	X	DEES	Western	Undisturbed	Spain	38°23'25.18"N, 3°29'39.88"W
X	X	HCES	Western	Disturbed	Spain	37º18'05.93"N, 5º57'57.81"W
X	X	HMES	Western	Disturbed	Spain	37º16'35.66"N, 5º57'16.80"W
X	X	IMES	Western	Disturbed	Spain	37º13'16.73"N, 5º58'30.50"W
X	X	MAES	Western	Disturbed	Spain	43º29'31.859"N, 3º31'28.808"W
X	X	GHIL	Eastern	Disturbed	Israel	32º10'30.5"N, 34º56'01.6"E
X	X	HZIL	Eastern	Disturbed	Israel	32º10'30.5"N, 34º49'28.9"E
X	X	REIL	Eastern	Disturbed	Israel	31°54.157'N, 34°49.407'E
X	X	TYIL	Eastern	Disturbed	Israel	32°15.284'N, 34°52.015'E
X	X	ZYIL	Eastern	Disturbed	Israel	31°56'00.4"N, 34°47'11.4"E
X	X	AUFI	Non-Native	Ag field	Finland	60°38'54"N, 22°33'53"E
X	X	BINY	Non-Native	Ag field	New York	42°11'2.4"N, 75°50'7.08"W
X	X	COAU	Non-Native	Ag field	Australia	31°18'S, 152°20'E
	X	KAMI	Non-Native	Ag field	Michigan, USA	42°21'34.6"N, 85°35'54.2"W
	X	MAFI	Non-Native	Disturbed	Finland	60°33'12"N, 22°06'53"E
	X	NAAU	Non-Native	Ag field	Austraila	36°96'S, 140°73'E
X		NCDE	Non-Native	Ag field	Germany	52°58.698'N, 9°37.865'E; alt: 224
X	X	WEAU	Non-Native	Ag field	Austraila	31°23'S, 118°32'E
R.pugioniformis						
X	X	GMIL	Native Range	Undisturbed	Israel	32°30'01.6"N, 35°24'51.4"E
R.s.convar.sativus						
	X	CGBC	Chinese Green Luobo	daikon	Baker Creek Heirloom	Cat#RD119
	X	FGBC	Formosa Giant Luobuo	daikon	Baker Creek Heirloom	Cat#RD127
X	X	MYJO	Miyashige	daikon	John Scheepers	625.11
X	X	NEJS	New Crown	daikon	John Scheepers	3860
X	X	TOBG	All Seasons Tokinashi	daikon	Bountiful Gardens	VRA-5050
X	X	WMBG	Watermelon	daikon	Bountiful Gardens	VRA-5100
X	X	CBBG	Cherry Belle	European	Bountiful Gardens	VRA-5080
X	X	DAJO	D'avignon	European	John Scheepers	620.11
X	X	ESNK	Early Scarlet Globe	European	NK Lawn & Garden Co	7576
	X	FRSI	Flamboyant Long Italian	European	GrowItalian.com	
	X	LBBC	Long Black Spanish	European	Baker Creek Heirloom	
	X	RABS	Raxe	European	Burpee's Signature Seeds	
	X	RBBC	Round Black Spanish	European	Baker Creek Heirloom	
X	X	SPNK	Sparkler	European	NK Lawn & Garden Co	7583
R.s.convar.oleifera						
X	X	ADOL	Adagio	Oilseed	MSU	
X	X	AROL	Arena	Oilseed	MSU	
X	X	COOL	Colonel	Oilseed	MSU	
	X	OIBG	Oilseed Radish	Oilseed	Bountiful Gardens	GRA-7378
R.s.convar.caudatus						
X	X	MABG	Madras	Rat tail	Bountiful Gardens	VRA-5060
X	X	RABG	Rat's Tail	Rat tail	Bountiful Gardens	VRA-5070
X	X	RAJS	Rat's Tail	CRat tail	John Scheepers	3870

Locus	Linkage	Cm	Contig	Enzyme	Forward Primer	Reverse Primer	Amp/Rep	Allele 1	Allele 2	Mg(mM)	Tm(C)	Anneal(C)
DWRD_124	1	0	CL427Contig8	HindIII	TGGCGGAAAGCAAAGAGAACTACG	AAAGGAAAGTCACAAGCGGTGCAG	400	250	150	2.0	58	72
Na12-E05	1	12			CGTATGTTTGTTCCACCTGC	ACTAGCAACCACAACGGACC	CA	133	150	2.5	55	52
DWRD_112	1	37	CL22Contig17	HindIII	TGACCTTGACCTTGATTCCGAGCA	ATGTTCTCGGTGAGAAAGGGAGGA	1200	1000	200	2.0	58	72
Na14-E08	1	48			TTACTATCCCCTCTCCGCAC	GCGGATTATGATGACGCAG	GCC	85	122	2.5	55	50
DWRD_61	1	54	CL2272Contig2	PstI	TAGTGGTTCGTCATCGGCTTCAGT	TAACTCACTTGTTGCCGGAGCAGA	700	600	100	2.0	58	72
DWRD_123	2	2	CL4189Contig2	HindIII	CCTTTGAGCTTGCGCTTTCCTTCT	TACCCACTTGGATGGCAGAAACCT	400	225	175	2.0	58	72
DWRD_107	2	16	CL1985Contig1	HindIII	AAACCGGTTCCATGAGAATGCCAC	ATCCTGGGCAGCTCAATTACCCAA	370	200	170	2.0	58	72
DWRD_177	2	46	CL2355Contig3	BcII	ATCATTCTCATCCTCAGTCGCCCT	AGTTTGTGAGGACGAGGCTTGACT	600	400	200	2.0	58	72
Ra1-H08	3	9			GTCGATGATCACGGAAGAGG	CTTGACAGCTACGGTTTGTCC	AGG/CGG	184	204	2.5	56	52
Bn26A	5	42			TAAACTTGTCAGACGCCGTTATC	CCCGTAAATCAAGCAAATGG	GA	90	120	2.5	53	52
BRMS-005	5	57			ACCTCCTGCAGATTCGTGTC	GCTGACCTTTCTTACCGCTC	GA	132	175	2.5	56	50
DWRD_121	6	11	CL370Contig1	HindIII	TCATCTTCCTCCTCGGTTGCTGAT	AGAAATCGACCGGATGGTGAAGGA	400	250	150	2.0	58	72
DWRD_48	6	23	CL1174Contig1	PstI	CACCACCGCCCAATCTCAACAAAT	TGGTATAGCAAGGGCAGCGTAAGT	600	400	200	2.0	58	72
Ra2-E11	6	53			GGAGCCAGGAGAGAAGAAGG	CCCAAAACTTCCAAGAAAAGC	CT	167	191	2.5	54	61
DWRD_158	6	65	CL3595Contig2	EcoRi	CAAGCCGCAGACCAAATCAACACT	GCTGCCGAGCTTGAACCAAACATT	400	250	150	2.0	58	72
DWRD_180	8	21	CL2638Contig1	BcII	ATCCAACTTGACGGTGTCAACGGA	AAAGGTGTTCGTGTCTGGCTAGGT	350	250	100	2.0	58	72
DWRD_205	9	20	CL1128Contig1	EcoRV	GTGGTTTCGAAGCTTTGTTTCTCCG	TAGTTGTCGGGAGGAAGCGTGATT	300	275	25	2.0	58	72
DWRD_27	9	35	CL126Contig10	NsiI	AGGTCCGGCTTCTCTAGTGATCTT	ACTCTGTCAAGTCATGCTTCGCGT	600	400	200	2.0	58	72
Na10-H06	S1				AGAATGAGACCCAGAAACCG	GCCACACTCTCTCTTACTAGGGC	CT	120	140	2.5	56	52
DWRD_97	S2		CL1509Contig2	HindIII	TGACGTGTAGTGTAGCGTTTGCGT	TGAACATAGAACCGACCACCTCCA	650	350	300	2.0	58	72
Bn35d	S3				GCAGAAGGAGGAGAAGAGTTGG	TTGAGCCGTAAAGTTGTCACCT	GA	222	251	2.5	58	61

Figure S2: PCR parameters and restriction enzymes for 21 SSR and CAPS markers. The allele 1 and 2 are the sizes of the two fragments for the CAPS and the size of the largest and smallest possible alleles for the SSRs.

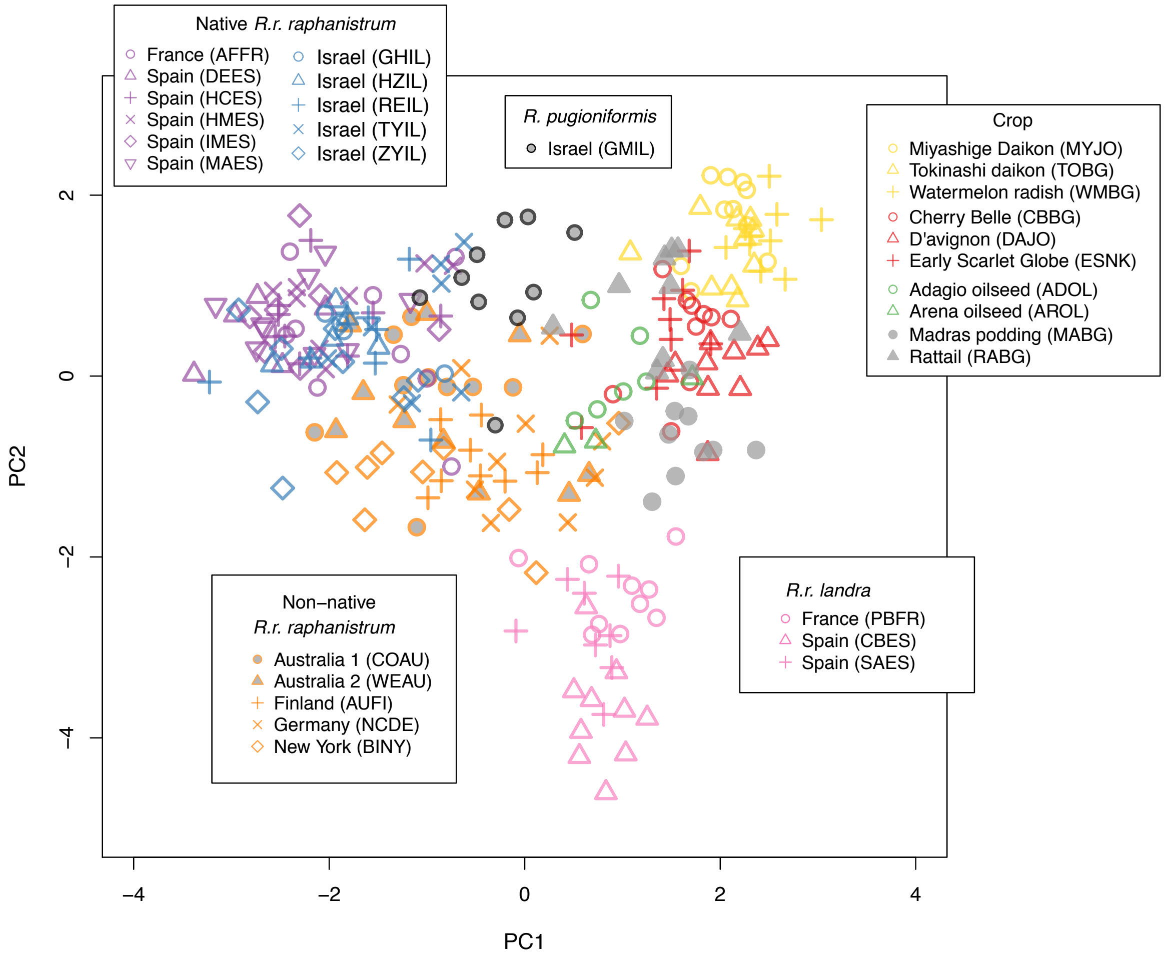

Figure S3: PCA plot of the first two eigenvectors of a principal components analysis of 30 *Raphanus* populations genotyped at presumed neutral markers. Each point is an individual, and each population is represented by 8-10 individuals. Populations are identified by plotting character and colored to match the SmartPCA figure in the main text (Fig. 2) This PCA was generated in R by running prcomp on the same dataset given to SmartPCA but with missing data omitted (na.omit). This PCA largely recapitulates the SmartPCA results, however some individuals are have been lost due to missing data

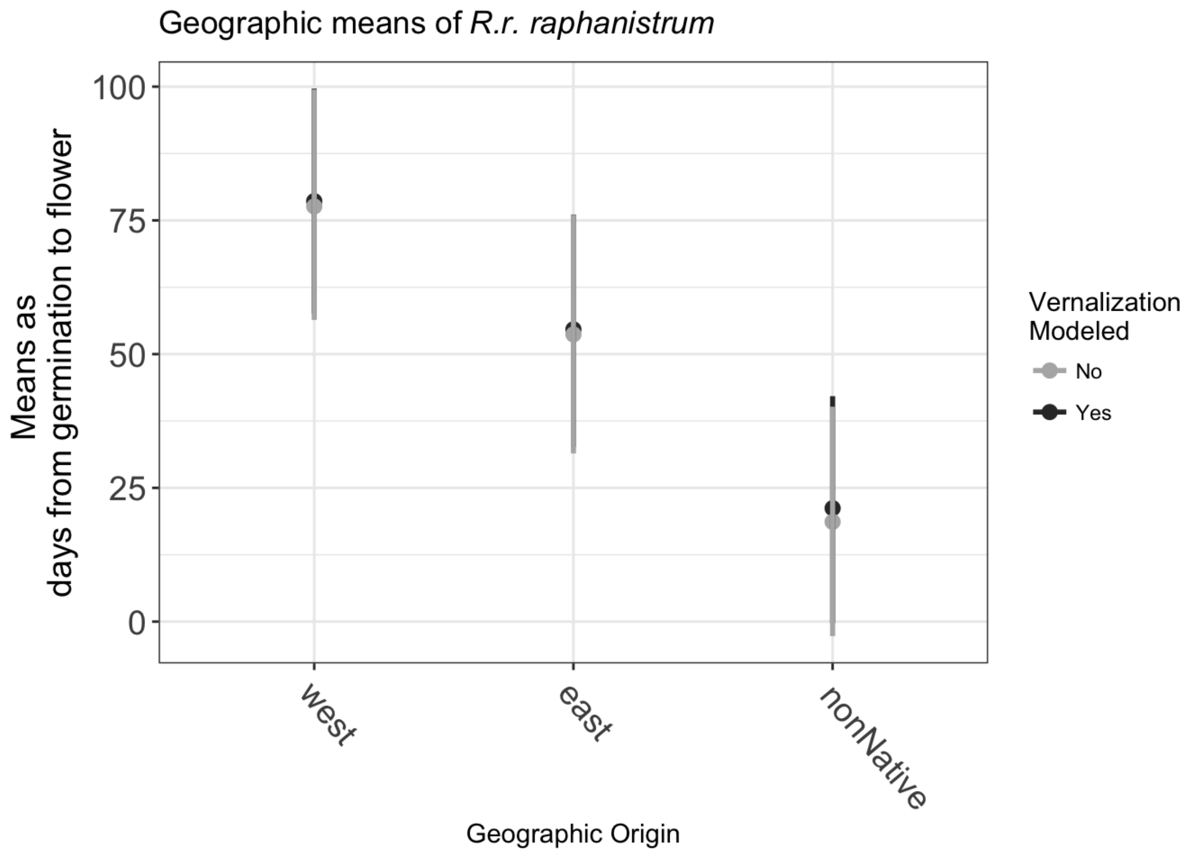

Figure S4: Geographic means of *R.r. raphanistrum* and 95% credible intervals for number of days from germination to flowering. ‘NonNative’ populations were collected outside of the Mediterranean region, all but one from agricultural fields (Table S1). ‘East’ and ‘West’ populations were collected either in Israel or in Spain or France, respectively. Removing the fixed effect of vernalization from the model had a negligible effect on geographic means.

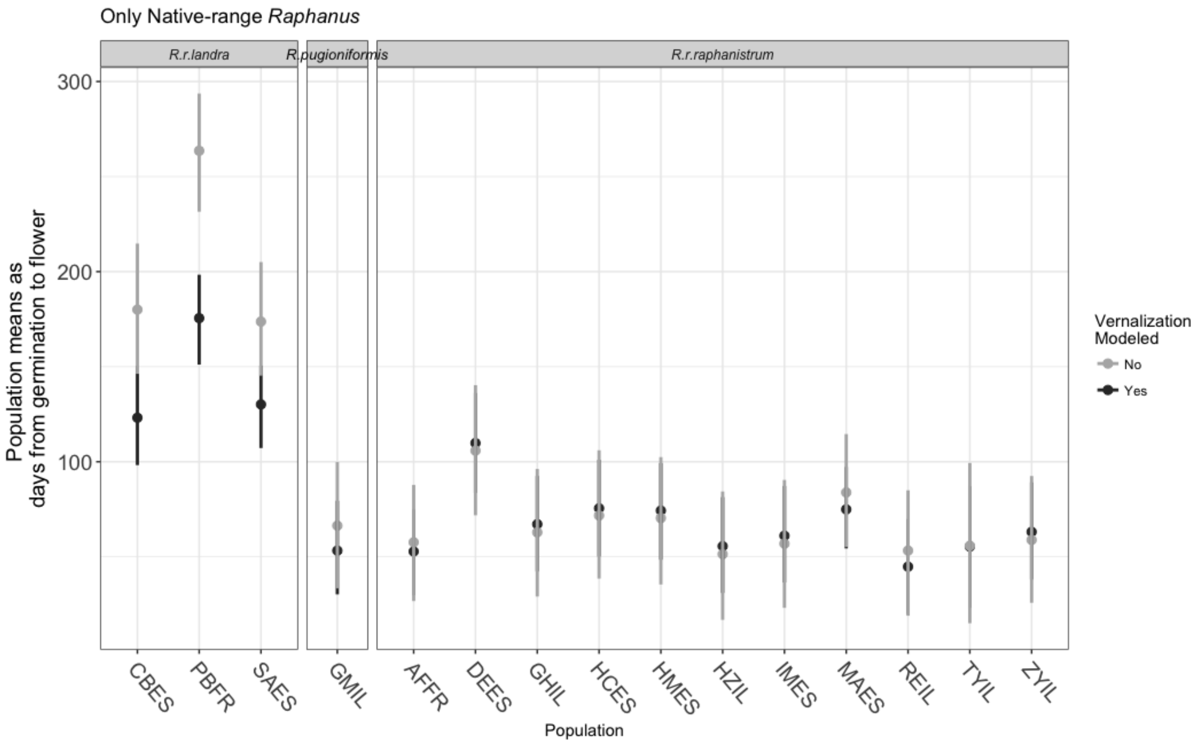

Figure S5: Native Range *Raphanus* population means and 95% credible intervals for number of days from germination to flowering for all *Raphanus* populations collected in the Mediterranean region. Accounting for differences in vernalization treatment had a negligible effect in most instances, however it significantly reduced the estimates for *R.r. landra* populations.(Population abbreviations as per Table S1)

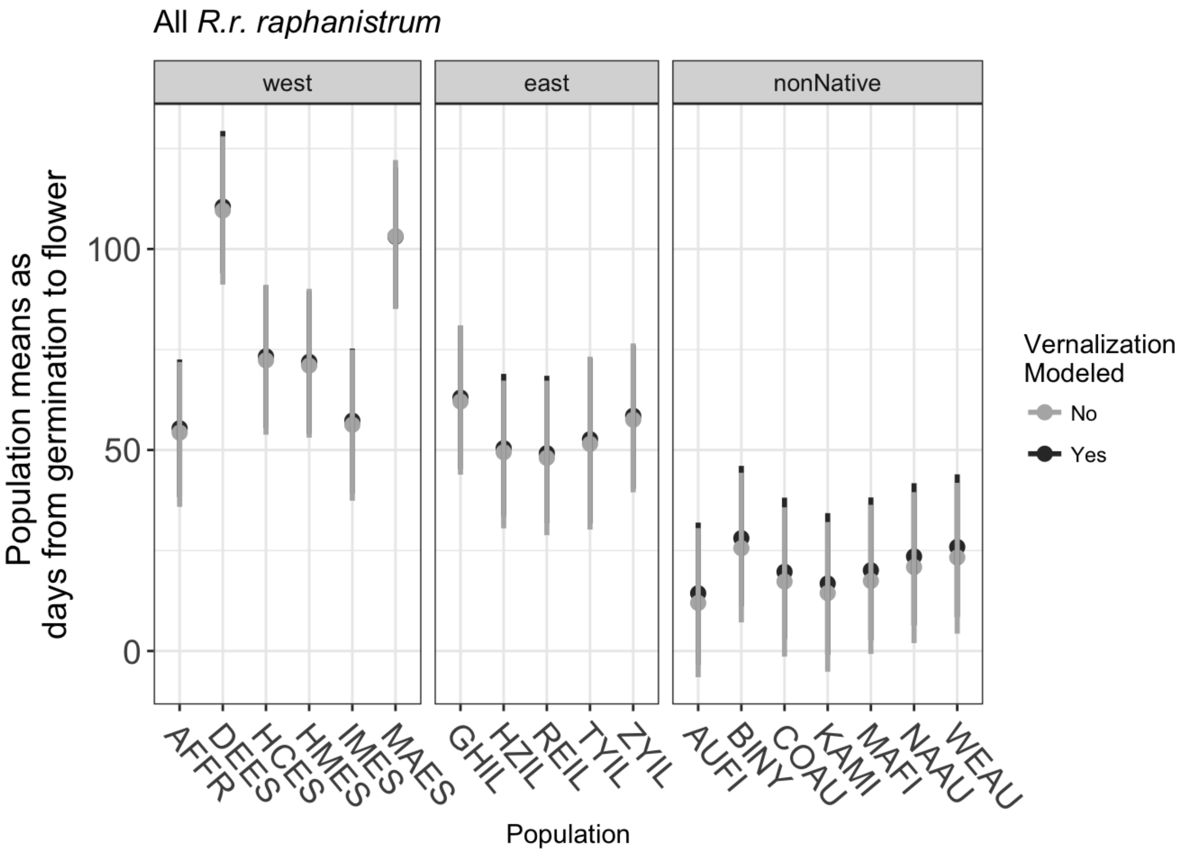

Figure S6: Population means of *R.r. raphanistrum* and 95% credible intervals for no. days from germination to flowering. 'NonNative' populations were collected from agricultural fields outside of the Mediterranean region. 'East' and 'West' populations were collected either in Israel or in Spain or France, respectively. Removing the fixed effect of vernalization from the model had a negligible effect on population means.

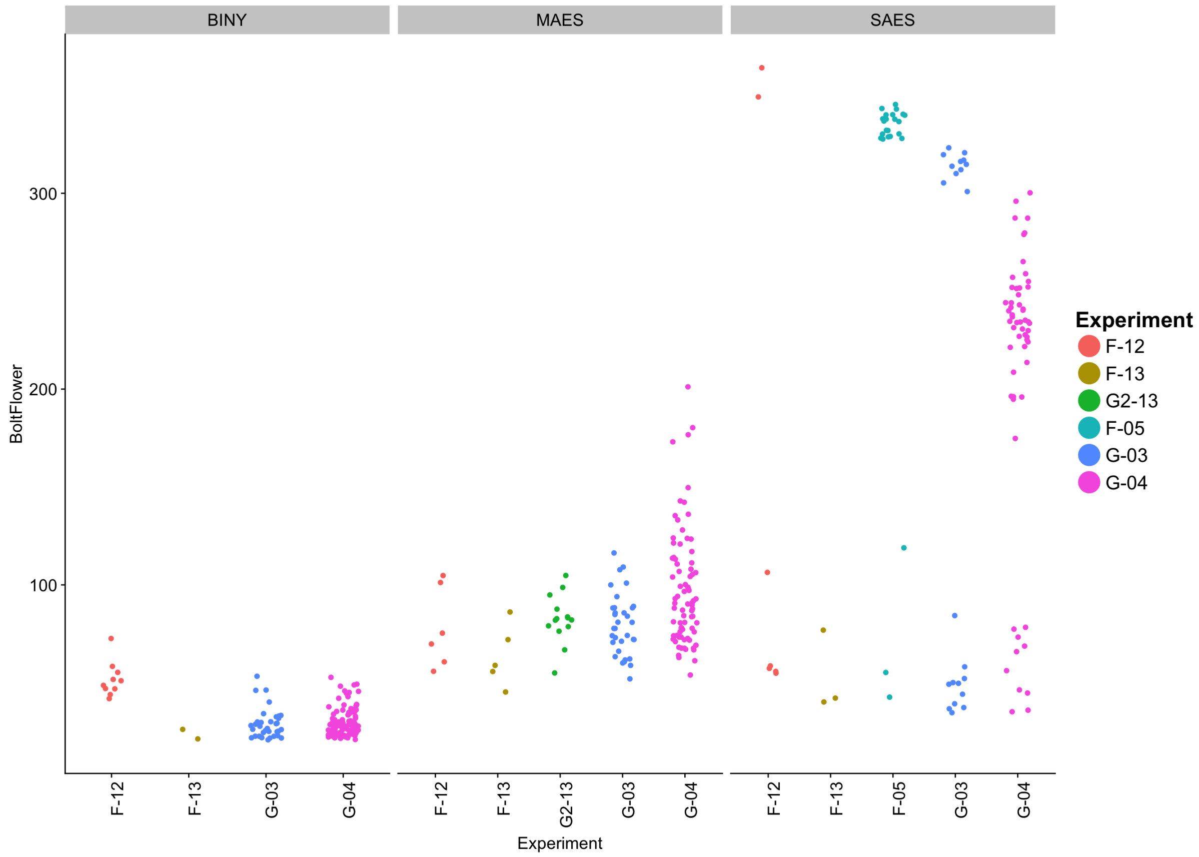

Figure S7: Raw flowering times of all individuals from three populations. BINY, a weed, shows little variation in flowering time within or between experiments. Both MAES and SAES show a large amount of within population variation in Figure 3, however very little of this variation appears to be due to experiment. SAES has very similar flowering time distributions in all experiments except F-13, where it appears they all flowered early. However, F-13 was a field experiment, and none of the SAES plants survived the winter, so individuals who were alive and would have likely flowered the following year are not included. Had they survived, SAES would have had a bi-modal flowering time distribution in all experiments

Figure S8: Selected summary statistics. Bolded lines are apriori grouping values and are preceded by population values for that group. 'Het' is the expected heterozygosity as calculated from all 21 markers. CoV is the coefficient of variation for days for flower, calculated as mean/standard deviation.

**Population**	**Het**	**CoV**
PBFR	0.34	2.50
CBES	0.21	56.9
SAES	0.28	50.7
R.r.landra	**0.35**	**44.0**
AFFR	0.41	45.0
DEES	0.36	17.1
HCES	0.43	18.8
HMES	0.45	19.1
IMES	0.43	28.2
MAES	0.41	29.3
WesternR.r.raphanistrum	**0.47**	**43.4**
GHIL	0.38	17.1
HZIL	0.42	28.1
REIL	0.42	28.9
TYIL	0.44	31.6
ZYIL	0.39	20.3
EasternR.r.raphanistrum	**0.45**	**27.8**
AUFI	0.42	12.7
BINY	0.50	28.8
COAU	0.38	29.6
KAMI		18.1
MAFI		13.9
NAAU		23.1
NCDE	0.47	
WEAU	0.48	24.1
WeedyR.r.raphanistrum	**0.52**	**25.7**
GMIL	0.36	24.8
R.pugioniformis	**0.36**	**24.8**
CGBC		27.7
FGBC		27.0
MYJO	0.33	37.4
NEJS	0.32	15.5
TOBG	0.26	18.2
WMBG	0.22	26.8
Daikon, R.s.convar.sativus	**0.36**	**42.0**
CBBG	0.28	24.6
DAJO	0.23	14.7
ESNK	0.26	26.7
FRSI		18.4
LBBC		24.8
RABS		26.3
RBBC		29.1
SPNK	0.24	36.8
European, R.s.convar.sativus	**0.33**	**32.0**
ADOL	0.34	31.9
AROL	0.39	19.2
COOL	0.44	21.3
OIBG		21.8
R.s.convar.oleifera	**0.45**	**31.7**
MABG	0.38	18.0
RABG	0.35	20.2
RAJS	0.38	30.1
R.s.convar.caudatus	**0.44**	**24.3**
